# Untranslated regions of diverse plant viral RNAs vary greatly in translation enhancement efficiency

**DOI:** 10.1186/1472-6750-12-22

**Published:** 2012-05-06

**Authors:** Krzysztof Treder, W Allen Miller

**Affiliations:** 1 Department of Plant Pathology & Microbiology, and Center for Plant Responses to Environmental Stresses, Iowa State University, Ames, IA, 50011, USA; 2 1615 E 8th St, #6, Davis, CA, 95616, USA

## Abstract

**Background:**

Whole plants or plant cell cultures can serve as low cost bioreactors to produce massive amounts of a specific protein for pharmacological or industrial use. To maximize protein expression, translation of mRNA must be optimized. Many plant viral RNAs harbor extremely efficient translation enhancers. However, few of these different translation elements have been compared side-by-side. Thus, it is unclear which are the most efficient translation enhancers. Here, we compare the effects of untranslated regions (UTRs) containing translation elements from six plant viruses on translation in wheat germ extract and in monocotyledenous and dicotyledenous plant cells.

**Results:**

The highest expressing uncapped mRNAs contained viral UTRs harboring Barley yellow dwarf virus (BYDV)-like cap-independent translation elements (BTEs). The BYDV BTE conferred the most efficient translation of a luciferase reporter in wheat germ extract and oat protoplasts, while uncapped mRNA containing the BTE from Tobacco necrosis virus-D translated most efficiently in tobacco cells. Capped mRNA containing the Tobacco mosaic virus omega sequence was the most efficient mRNA in tobacco cells. UTRs from Satellite tobacco necrosis virus, Tomato bushy stunt virus, and Crucifer-infecting tobamovirus (crTMV) did not stimulate translation efficiently. mRNA with the crTMV 5′ UTR was unstable in tobacco protoplasts.

**Conclusions:**

BTEs confer the highest levels of translation of uncapped mRNAs in vitro and in vivo, while the capped omega sequence is most efficient in tobacco cells. These results provide a basis for understanding mechanisms of translation enhancement, and for maximizing protein synthesis in cell-free systems, transgenic plants, or in viral expression vectors.

## Background

Plant RNA virus genomes are among the most efficiently translated mRNAs known. After invading plant cells, positive strand RNA viruses employ diverse strategies to take over the host translational machinery. All host cellular mRNAs and some viral RNAs have a 5′ cap to recruit the translational machinery via binding of the eIF4E subunit of translation initiation factor complex eIF4F [1,2]. Many plant viral RNAs lack a 5′ cap and have either an internal ribosome entry site (IRES) in the 5′ untranslated region (UTR) or a cap-independent translation element (CITE) in the 3′ UTR to facilitate translation (reviewed in [3-6]). Many CITEs bind translation initiation factors, including one or more subunits of eIF4F [6-9] which, in plants, is a heterodimer of the cap-binding protein eIF4E and the scaffolding protein eIF4G [10]. In most cases the 3′ CITE must base pair to the 5′ UTR, presumably to deliver initiation factors to the 5′ end where the ribosome is recruited and translation initiates [11-14]. Many viral RNAs also lack the poly(A) tail that is required for efficient translation initiation. Instead they have evolved structures that replace the need for a poly(A) tail [15,16].

**Figure 1:**
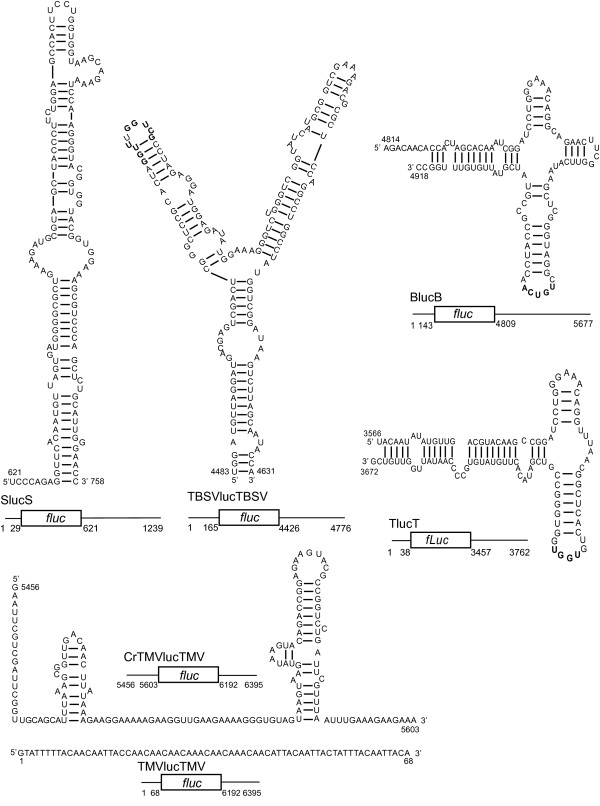
**Maps of mRNAs and secondary structures of translation elements in the UTRs.***fluc* is the firefly luciferase ORF, flanked by viral UTRs. Above each construct is the secondary structure and sequence of the translation element in that UTR. TMV omega and crTMV elements are located in the 5′ UTR, the others are in the 3′ UTR. The numbers indicate the genomic coordinates of UTRs and the translation elements. Bold text indicate bases shown to base pair to the 5′ UTR.

At least half of plant viruses have uncapped genomic RNAs that are translated efficiently. The genomic RNAs of *Barley yellow dwarf virus* (BYDV), *Tobacco necrosis virus D* (TNV-D), *Satellite tobacco necrosis virus 1* (STNV-1) and *Tomato bushy stunt virus* (TBSV) have no cap or any other known modification at the 5′ end and they lack a poly(A) tail. BYDV and TNV-D RNAs harbor a BYDV-like CITE (BTE) in the 3′ UTR, defined by a conserved 17-nt sequence that forms part of a set of helices protruding from a central hub (Figure 1) [13,17-19]. STNV-1 RNA contains a completely different type of 3′ CITE, called the translation enhancer domain (TED), consisting of a stem-loop with multiple bulges [20-23]. The TBSV 3′ UTR has a third type of 3′ CITE that forms a Y-shaped structure (YSS) which is conserved in many members of genus *Tombusvirus*[12,14,24]. These three types of CITE are unrelated in sequence and structure but all have potential to base pair to the 5′ UTR via kissing stem-loops (Figure 1). This base pairing is required for the BYDV BTE and Y-shaped structures [11,12] but is reported not to be required for the BTE of Red clover necrotic mosaic virus (RCNMV), called TE-DR1 [25] or for the STNV-1 TED [26].

Crucifer-infecting tobamovirus (crTMV) has a capped genomic RNA, but the coat protein ORF near the 3′ end of the genome is translated via an internal ribosome entry site (IRES) [27]. This IRES, rich in GAAA repeats, was reported to promote very efficient cap-independent translation of reporter genes in plant and mammalian cells [28]. The genomic RNA of *Tobacco mosaic virus* (TMV, genus Tobamovirus) contains a 5′ cap and is translated very efficiently due to a 5′-leader sequence (Ω) that has few guanosine residues [29,30].

None of the above RNAs contains a poly(A) tail. TMV and TNV-D genomes have different highly structured 3′ ends that functionally substitute for the poly(A) tail [15,16]. The sequences in the other viruses that permit poly(A) tail-independent translation are not well characterized.

Translation enhancer elements, as parts of transgenes, or in viral expression vectors, are useful for expressing high levels of protein in plant cells [4,30,31] For biotechnological applications intended to maximize protein expression, it would be valuable to know which translation elements stimulate translation to the highest levels. Also, while studies on mechanisms of these elements have been published, understanding how active each element is relative to the others would reveal the relative efficiencies of the different mechanisms that have been reported. Because side-by-side comparisons of these translation elements are lacking, here we directly compare the translational stimulatory effects of viral UTRs harboring the translation elements of the six viruses discussed above. We compare translation of mRNAs containing the complete 5′ and 3′ UTR of each virus (except for crTMV, where we used the 3′ UTR of TMV) in vitro and in vivo. The minimal sequence of each element has been defined, and each is confined within a UTR, but rather than use only the minimal translation enhancer as a UTR, we included the full viral UTRs because sequence outside the translation element is often needed for full translation activity in vivo, including for example poly(A) tail “substitute” sequences that are needed in addition to the 3′ CITE for translation in vivo [16]. Using these constructs, we found surprisingly wide variations in efficiency of translation mediated by the translation elements of the six viruses.

## Results and discussion

All constructs consisted of the firefly luciferase ORF flanked by the complete UTRs of each virus (Figure 1). This ensured that the 5′ and 3′ UTRs were compatible and complementary in the BYDV, TNV-D and TBSV constructs in which the 3′ CITE must base pair to the 5′ UTR for efficient translation initiation. The omega sequence of TMV and the crTMV IRES are both located in 5′ UTRs. Because we had no access to crTMV virus, we paired the 3′ UTR of TMV with the 5′ UTR of crTMV, which was synthesized. Both viruses are in the same genus and have similar 3′ UTRs [32]. Importantly, the crTMV IRES was shown previously not to require a specific 3′ UTR sequence in order to function in vitro or in vivo [28]. The 3′ UTR of TMV was shown previously to facilitate efficient translation in vivo, as it contains a pseudoknot-rich repeat region and a terminal tRNA-like structure that obviate the need for a poly(A) tail [15,33]. Translation of capped and uncapped transcripts was compared in the widely used, efficient and high fidelity wheat germ extract, and in monocot (oat) and dicot (tobacco) protoplasts.

### Kinetics of translation in vivo

**Figure 2:**
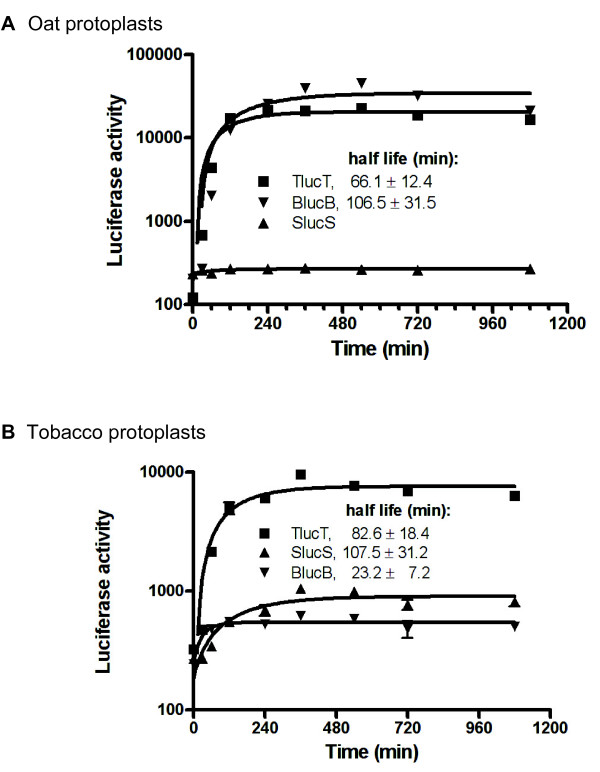
**Time-course of luciferase activity from selected uncapped mRNAs in protoplasts.A**, oat protoplasts. **B**, tobacco protoplasts. Firefly luciferase activity was measured at the indicated time points. Functional half-lives (+/− standard error) were calculated as described in Methods.

To determine the appropriate time points at which to compare translation efficiencies of mRNAs, luciferase expression from the three constructs derived from STNV-1, TNV-D and BYDV (named SlucS, TlucT and BlucB, respectively) was measured at different times post electroporation in oat and tobacco protoplasts. The luciferase levels increased during the first 6 hours after electroporation (Figure 2). We conclude that, in subsequent experiments, assaying protoplasts at 4 hours post electroporation would provide an accurate indication of the translation efficiency of the various constructs to be tested.

### 5′ cap structure increases translation in vitro

**Figure 3:**
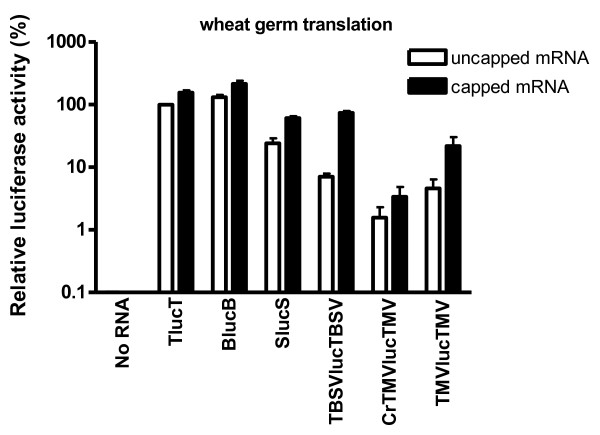
**Translational activity of indicated Luc mRNAs in wheat germ extract.** The relative luciferase activity (initially measured as relative light units, rlu) of uncapped (white bars) and capped (black bars) transcripts is presented as a percentage of the rlu obtained from uncapped TlucT mRNA. Error bars show the standard errors. The assays were performed in three independent experiments in which each mRNA was tested in triplicate.

To avoid complications involved in in vivo (protoplast) assays due to differences in electroporation efficiency, cell survival, RNA stability differences and RNA access to ribosomes, we compared translation activities in wheat germ extract. Luciferase activity obtained with uncapped TlucT was defined as 100%, because this RNA always gave high translation levels. Of the uncapped RNAs, BlucB translated about 25% more efficiently than TLucT, SLucS was about one-fourth as efficient, while the other uncapped RNAs translated less than 10% as much as TLucT (Figure 3, note the log_10_ scale). The presence of a cap stimulated TlucT and BlucB translation by about 50%, in agreement with previous reports [17,19]. SlucS was stimulated about 2.5-fold by a cap, similar to the result reported by Timmer et al. [22] who compared the translation of uncapped and capped mRNA from a construct containing an α-globin ORF flanked by STNV-1 5′ and 3′ UTRs.

Our in vitro analysis of reporter RNA containing TBSV UTRs (TBSVlucTBSV) demonstrated that uncapped RNA was 7% as efficient as TlucT, and capping increased translation about 10-fold. Similar results were also shown by Wu and White [34], who observed that uncapped defective interfering RNA (DI-RNA), containing TBSV 5′ UTR, 3′ CITE and *GUS* gene, was not functional in wheat germ extract, while the addition of a cap increased translation about 5-fold above background.

Presence of a cap stimulated translation of the reporter with TMV UTRs (TMVlucTMV) about 5-fold, supporting previous observations of cap-dependent enhancement of translation by the omega sequence in the 5′ UTR of TMV [33]. Surprisingly, translation of both capped and uncapped mRNAs containing the reported crTMV IRES sequence (crTMVlucTMV) was extremely low. Overall, the differences in translation efficiency in wheat germ extract imposed by various plant virus UTRs reported to be translation enhancers are striking.

### UTRs containing translation enhancers behave differently in oat and tobacco cells

**Figure 4:**
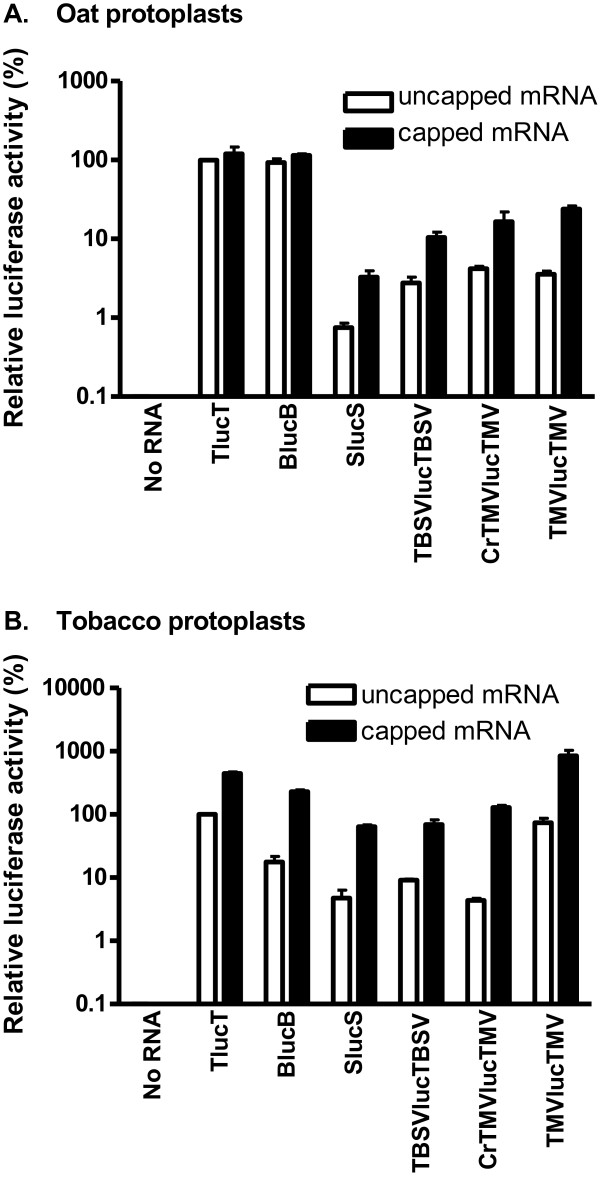
**Expression of uncapped and capped luciferase mRNAs in protoplasts.A**, oat protoplasts. **B**, tobacco protoplasts. Relative luciferase activities from indicated uncapped transcripts (white bars) and capped transcripts (black bars) were measured 4 h after electroporation, and shown as percentages of uncapped TlucT activity.

BYDV infects only moncotyledonous plants such as oat, wheat and other grasses, while the other viruses are limited to dicots such as tobacco and do not infect wheat from which the extract was obtained for in vitro analysis. To investigate whether the translation stimulating activity of viral UTRs varies between monocots and dicots, we compared translation of the reporter constructs in oat and tobacco protoplasts. Of the uncapped transcripts in oat protoplasts, TlucT and BlucB yielded the highest luciferase expression, while uncapped transcripts of TBSV, crTMV and TMV yielded very low levels (Figure 4A). Addition of a 5′ cap increased translation of TlucT and BlucB about 1.5 fold, in agreement with published results [17,18,35]. For Luc mRNAs with TBSV UTRs, presence of a cap increased translation 3-fold in oat protoplasts (Figure 4A). The stimulatory effect of cap structure was demonstrated originally in cucumber protoplasts [34]. The uncapped SlucS mRNA yielded less than one-hundredth of the luc activity as TlucT or BlucB. Even the capped version of this RNA translated with low efficiency.

Strikingly, translation of all mRNAs was far more cap-dependent in tobacco than in oat protoplasts. Addition of a cap increased translation efficiency of all constructs by at least four-fold (Figure 4B). The cap enhanced translation of Luc mRNAs with TMV and crTMV 5′ UTRs by more than 10-fold (Figure 4B). This level of stimulation by capping of TMV omega-containing mRNAs was shown previously [33]. The reporter with TMV UTRs was stimulated 12-fold by a cap, but the omega sequence stimulated translation so efficiently that even the uncapped version was 74% as efficient as uncapped TLucT, the mRNA that exhibited the highest translation efficiency among all uncapped mRNAs tested in this experiment. In tobacco protoplasts, uncapped BLucB mRNA yielded only 18% as much luciferase as TLucT (Figure 4B). The other uncapped mRNAs were less than 10% as efficient as TlucT. Relative to BLucB, the UTRs from tobacco-infecting viruses all stimulated more efficient translation compared to oat protoplasts, but expression of the uncapped STNV, TBSV, and crTMV-derived RNAs was still lower than that of BLucB.

### Luc mRNAs differ in stability

To determine contribution of mRNA stability to the differences in luciferase expression, we determined the functional and physical stabilities of selected mRNAs. We used the data in Figure 2 to determine functional stability of actively translated mRNAs by calculating their functional half-lives using an equation that describes protein accumulation as a function of time [21], rewritten to allow for the calculation of both the translation efficiency of mRNA and its half-life [26]. The functional half-lives in tobacco and oat protoplasts, respectively, were 83 and 66 min for TLucT, and 23 and 106 min for BlucB. The functional half-life of SLucS was 107 min in tobacco protoplasts, but its luciferase expression was too low to calculate a functional half-life in oat cells (Figure 2). TLucT is the most efficient uncapped mRNA in tobacco protoplasts and is nearly as efficient as BLucB in oat protoplasts. SLucS is the least efficient mRNA in both systems. In general, oat protoplasts create a less stringent translation environment, both in terms of higher mRNA stability, and translational efficiency for TLucT and BLucB.

**Figure 5:**
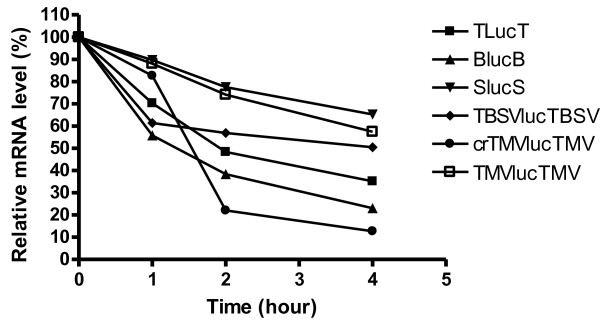
**Stabilities of luc mRNAs in tobacco protoplasts.** Uncapped luc reporter mRNAs containing the indicated UTRs were electroporated into tobacco protoplasts. Real-time qRT-PCR was performed as described in the Methods. The relative amount of each transcript at 0 hr was defined as 100%.

Physical stability was measured in tobacco protoplasts by using real-time qRT-PCR to quantify mRNA levels over time [36-38]. As seen with functional stabilities, the half-lives of the mRNAs varied greatly, and they did not always correlate with luciferase levels. Among all RNAs, crTMV Luc mRNA was the least stable, with only 13% remaining 4 hr after electroporation. In contrast, 65% of SlucS Luc mRNA remained, agreeing with its relatively high functional stability. The half-lives of SlucS and TMVlucTMV RNAs were greater than 4 hr. The half-lives of the other RNAs were calculated as: TBSVlucTBSV t_1/2_ = 240 min; TlucT t_1/2_ = 115 min; crTMV-luc-TMV t_1/2_ = 92 min and BlucB t_1/2_ = 77 min (Figure 5). The physical half-life of TLucT was similar to the functional half-life, but the physical half-lives appeared longer for SlucS and BlucB. This may be due to partially degraded mRNA and mRNA that was not available to ribosomes, but remained detectable by qRT-PCR. These mRNA populations would not be detected in functional half-life assays. We speculate that the unusual nonlinear curves for crTMV and TBSV RNAs are due to the RNA existing in more than one conformation, with each conformation having a different stability. Note that the TMV and crTMV RNAs differ only in their 5′ UTRs, thus the 5′ UTR of TMV (the omega sequence) appears to confer substantially more RNA stability than the GAAA-rich IRES in the crTMV 5′ UTR which apparently induces rapid degradation. Instability of crTMVlucTMV RNA may be explained by its natural role as an internally located IRES. This internal location may protect it from cellular 5′ to 3′ exonuclease activity, while the other 5′ UTRs used in this study are naturally located at the 5′ end and thus likely to have evolved structures that reduce susceptibility to exonucleases.

The instability of the mRNA with the crTMV 5′ UTR may explain the low expression levels to some extent, but the BlucB and TlucT mRNAs also were not the most stable mRNAs yet they generated more luciferase activity in tobacco protoplasts than any of the other uncapped mRNAs, except uncapped TMVlucTMV. When stability differences are accounted for, translation initiation on TLucT mRNA appears to be more efficient than on uncapped TMVlucTMV.

## Conclusions

We draw the following conclusions.

1. Viral UTRs vary by orders of magnitude in efficiency of facilitating translation in wheat germ extract and in protoplasts.

2. The UTRs containing BTEs (BYDV and TNV-D) conferred more efficient cap-independent translation than UTRs containing the other 3′ translation elements.

3. The TMV omega sequence gave much more efficient translation in tobacco than in oat cells, relative to the other RNAs, and the mRNA with capped omega sequence was the highest-expressing mRNA in tobacco cells.

4. The UTRs from monocot-infecting BYDV yielded higher expression in monocot systems, relative to the UTRs from dicot (tobacco)-infecting viruses, suggesting a correlation between virus host range and translation efficiency.

5. The TNV BTE is more efficient than the STNV TED sequence. This observation rules against the hypothesis that STNV parasitizes TNV due to its being a more translationally efficient mRNA. We propose that instead, STNV RNA outcompetes TNV RNA for the viral replication machinery, which would make STNV a less efficient mRNA because of antagonism between translation and replication of viral RNA.

6. Translation is more cap-dependent in protoplasts than in wheat germ extract, in agreement with previous observations of the TNV BTE [17,19]. Also it is more cap-dependent in tobacco relative to oat protoplasts (Figure 4). This may be due to different levels of eIF4F (or its subunits) in oat vs tobacco, or more efficient mRNA recognition and/or scanning by the 40 S ribosome in oat compared to tobacco.

This work provides perspective for future studies on relative efficiencies of the mechanisms of the translation elements investigated here. For example, these elements appear to interact differently with translation initiation factors [7,8,39]. The BTE binds directly to eIF4G and not eIF4E, although presence of eIF4E slightly enhances the BTE-eIF4G interaction [8]. Other CITEs appear to depend on both eIF4G and eIF4E [7,8,39,40]. This ability to stimulate translation in the absence of eIF4E may explain why BTEs confer the most efficient cap-independent translation of the translation elements studied here.

The comparisons reported in this work have biotechnological relevance. Large scale wheat germ extracts are being adapted for high throughput, massive production of proteins, without the limitations of the cell [41], for structural and other studies [42,43]. Addition of a cap-independent translation element to the mRNA encoding a desired protein, obviates the need for capping large amounts of mRNA which is expensive and reduces yield in transcription. Of the elements tested here, the BYDV BTE and UTR sequences appear to be the most effective cis-acting sequences for maximal translation in wheat germ extract, while the TNV BTE and UTRs provide most efficient cap-independent translation in tobacco cells. These elements may also be useful for high level expression of genes in transgenic plants, and for plant virus expression vectors [44-46].

## Methods

### Plasmid construction

pTlucT containing UTRs of TNV-D (GenBank ID: D00942) was constructed by Shen and Miller [17]. pBlucB, formerly called pLUC869, containing UTRs of BYDV (GenBank ID: X07653) was constructed by Wang et al. [47]. In pSlucS, pTBSVlucTBSV and pTMVlucTMV, reporter firefly luciferase gene was flanked by 5′ and 3′ UTRs of STNV-1 (GenBank ID: L06057), TBSV (GenBank ID: M21958) and TMV (GenBank ID: V01408), respectively. In pcrTMVlucTMV, the firefly luciferase gene was flanked by the IRES of crTMV (GenBank ID: Z29370) and the 3′ UTR of TMV.

Standard molecular techniques used to make constructs were as described in Sambrook and Russell [48]. The primers used for plasmid construction are listed in Table 1. Purification of DNA fragments by agarose gel electrophoresis was accomplished with the Qiagen Gel Extraction Kit. Plasmids were purified with the Qiagen Plasmid Miniprep Kit. All clones were sequenced at the Iowa State University DNA Sequencing Facility.

**Table 1:** Primers and sequences used in making plasmid constructs

*Primer*	*Sequence (5′ → 3′)*
GEM-T7-STNV	caggcggccgctaatacgactcactataggGAGTAAAGACAGGAAAC
Luc340R	ctgttgagcaattcacgttc
STNV-luc	GGGAGTAAAGACAGGAAACTTTACTGACTAACCatggaagacgccaa
STNV-3′F1	CCGCTTGAAGTCTTTAATTAAATAC
STNV-3′F2	CCAAATTGtaagcttctcgagCCCAGAGGTTCACAATG
STNV-3′R1	CCTCTGGGActcgagaagcTTACAATTTGGACTTTCCG
STNV-3′R2	CCAGGTATAGTTCTACAgttaacccggg
TBSV-3′F	gggctcgagGTTTGTGGAGATGAGTGT
TBSV-3′R	ggggttaacGGGCTGCATTTCTGCAA
TBSV-5′F	Gacggcggccgctaatacgactcactatagg
TBSV-5′R	cccgccatggTCGCTTGTTTGTTGGAA
TMV-3′F	gggcaagcttGGTAGTCAAGATGCATAA
TMV-3′R	tttcccgggTGGGCCCCTACCGGGGGTAA

The sequence is in 5′ → 3′ direction. The underlined sequences are recognized by restriction enzymes. Nucleotides in lowercase are non-viral sequences. Nucleotides in uppercase are viral derived sequences.

A plasmid containing cDNA of STNV-1 was a gift from Dr. Karen Browning (University of Texas at Austin). By using primers GEM-T7-STNV and Luc340R, the STNV-1 5′ UTR was amplified from the template, a PCR product generated from pBlucB by primers STNV-luc and Luc340R. The STNV-1 5′ UTR was then cut with Xba I and Eag I and ligated into the Xba I/Eag I sites of plasmid pBlucB, generating the intermediate plasmid pSlucB, which contains STNV-1 5′ UTR and BYDV 3′ UTR. pSlucB was manipulated to obtain pSlucS harboring STNV-1 5′ UTR and 3′ UTR as follows. The STNV-1 3′ UTR was amplified by overlapping PCR. The left half of the 3′ UTR was achieved by amplifying pBlucB with primers STNV-3′ F1 and STNV-3′ R1. The right half of the 3′ UTR was achieved by amplifying STNV-1 plasmid with primers STNV-3′ F2 and STNV-3′ R2. The full length 3′ UTR was amplified by overlapping PCR with the left half and right half of 3′ UTR, and the resulting full length 3′ UTR product was excised with Pac I and Sma I. The cassette was cloned into the Pac I/Sma I sites of the intermediate plasmid pSlucB, generating pSlucS.

The viral genomic 5′ UTR of crTMV was synthesized into pZERO2 plasmid by Integrated DNA Technologies (Coralville, IA). The 5′ UTR was flanked by a Not I site and T7 promoter sequence at the 5′ end and an Nco I site at the 3′ end. Plasmid pZERO2 was digested with Not I and Nco I, and cloned into the Not I/Nco I site of plasmid pSlucS, resulting in pcrTMVlucS. The TMV 5′ UTR was cut out from a plasmid containing TMV omega sequence and ligated into Not I/Nco I-cut pSlucS, creating an intermediate plasmid pTMVlucS. The TMV 3′ UTR was amplified by reverse transcription PCR of TMV viral RNA. TMV virions were provided by Dr. John Hill at Iowa State University. Viral RNA was isolated from purified virions by the SDS-phenol method [49]. Viral cDNA was synthesized by reverse transcription with primer TMV-3′ R. The TMV 3′ UTR was amplified from viral cDNA with primers TMV-3′ F and TMV-3′ R. The product was cut by Hind III and Sma I and ligated into the Hind III/Sma I site of pcrTMVlucS or pTMVlucS. The resulting plasmids were named pcrTMVlucTMV or pTMVlucTMV.

To generate TBSV UTRs, plasmid pTBSV-100 [50] was amplified with primers TBSV-5′ F and TBSV-5′ R, and with primers TBSV-3′ F and TBSV-3′ R. The two PCR products were ligated into Not I/Nco I and Xho I/Hpa I sites of plasmid pSlucS, creating pTBSVlucTBSV.

### In vitro transcription

All mRNAs were transcribed from plasmids linearized with SmaI. Uncapped and capped RNAs were synthesized using the T7 MegaScript, and mMESSAGE mMACHINE kits (Ambion), respectively. Integrity of transcripts was confirmed by agarose gel electrophoresis.

### In vitro translation

In vitro translation was conducted by adding 0.4 pmol of RNA transcript to 50 μl wheat germ extract (Promega) based on the manufacturer’s instructions. After two hours incubation at room temperature, luciferase activity was measured using the Dual Luciferase Reporter system (Promega) on a Glomax 20/20 luminometer (Promega). Each assay was repeated at least three times.

### In vivo translation

Tobacco NT1 protoplasts and oat protoplasts were prepared from cell suspension as described [51,52]. The electroporation and luciferase assays were performed as described [13]. The firefly luciferase activity for each construct was normalized against *Renilla reniformis* luciferase activity generated from the control transcript, RlucA [17], that was co-electroporated into protoplasts along with the luciferase-encoding test constructs. RlucA is a capped, polyadenylated transcript encoding *Renilla* luciferase and lacking viral sequences. The relative luciferase activity of uncapped TlucT mRNA was set as 100%. The relative luciferase activities of other constructs were presented as percentages of TlucT.

### Functional stability of mRNAs

Functional half-lives of mRNAs were determined according to Meulewaeter et al. [26] using this equation describing protein accumulation (P) as function of time (t):

(1) $$\text{P}(\text{t})=(A\cdot {\text{t}}_{1/2}/\text{ln}2)(1-{\text{e}}^{-(\text{ln}2/\text{t}1/2)(\text{t}-\text{T})})$$

*A* represents translational efficiency of mRNA (number of protein molecules translated from mRNA molecule per time unit) when the input of translatable mRNA is equal under all conditions. T corresponds to the time point at which the first protein molecule is completed and t to the assay time point. The functional half-life of mRNA (t_1/2_) is defined as the time in which the protein accumulation rate equals half the maximal rate and thus measures the stability of the actively translated mRNA. Values for *A,* t_1/2_ , and T were obtained by nonlinear regression using Equation (1) in the GraphPad Prism 4.0 software to calculate a best fitting curve to the experimental data points.

### Real-time quantitative PCR analysis of relative quantity of mRNA in tobacco cells

Tobacco NT1 protoplasts were electroporated with 10 μg uncapped transcripts of various constructs and incubated at room temperature. Cells were harvested at different times after electroporation and washed 3 times with 0.4 M mannitol. Total RNA was isolated from cells by RNeasy Mini Kit (Qiagen) and the residual amounts of DNA were removed by RNase-Free DNase Set (Qiagen) according to the manufacturer’s instructions. By using random hexamers, single strand cDNA was synthesized from 3 μg total RNA by Invitrogen SuperScript III first-strand synthesis kit, according to the manufacturer’s instructions. cDNA was used in the amplification reaction directly after dilution. Primers, LUCF1 (5′-GGCGCGTTATTTATCGGAGTT-3′) and LUCR1 (5′-TTCATACTGTTGAGCAATTCACGTT-3′), were designed specifically for Luc cDNA. The amplification reaction was performed on an Applied Biosystems 7300 real-time PCR system using SYBR green as a fluorescent dye. Each reaction was performed in a final volume of 50 μL with the following components: 5 pmoles of each primer, cDNA corresponding to 30 ng input RNA in the reverse transcription reaction, 25 μL of Power SYBR Green Mix (Applied Biosystems), and water to a final volume of 50 μL. The thermal cycle conditions were 50^0^ C for 2 min, 95^0^ C for 10 min, followed by 40 cycles of amplification (95^0^ C for 15 s and 60^0^ C for 1 min). Agarose gel electrophoresis was performed after each real-time quantitative PCR reaction to assess the presence of a unique final product. Amplification of 18 S rRNA was used as an internal control for expression of a housekeeping gene with the primers, 18 S rRNAF (5′ACTACGTCCCTGCCCTTTGTAC- 3′) and 18 S rRNAR (5′-GAACATTTCACCGGATCATTCAA- 3′). Fold changes in Luc mRNA abundance were calculated based on the relative quantification analytical method (2^−∆∆CT^) using 18 S rRNA amplification as internal standard. The results presented are averages of technical triplicates and represent at least two independent experiments. The relative quantity of RNA at 0 hr was set at 100%. The RNA quantity at 1 hr, 2 hr and 4 hr was determined as percentages of quantity at 0 hr. Half-life of Luc mRNAs was calculated as described in Leclerc et al. [36].

## Abbreviations

BTE = Barley yellow dwarf virus-like cap-independent translation element; BYDV = Barley yellow dwarf virus; CITE = Cap-independent translation element; crTMV = Crucifer-infecting tobamovirus; IRES = Internal ribosome entry site; ORF = Open reading frame; STNV = Satellite tobacco necrosis virus; TBSV = Tomato bushy stunt virus; TMV = Tobacco mosaic virus; TNV = Tobacco necrosis virus; UTR = Untranslated region.

## Competing interests

WAM is a co-patent holder on the BTE, US Patent No. 5,910,628. He receives no compensation based on sales or use of this RNA sequence.

## Authors’ contributions

QF designed experiments and performed all experimental work. KT calculated mRNA half-lives. WAM designed experiments and conceived the study. All authors wrote the manuscript. All authors read and approved the final manuscript.
